# Low-Cost Laparoscopic Skill Training for Medical Students Using Homemade Equipment

**DOI:** 10.15766/mep_2374-8265.10810

**Published:** 2019-02-27

**Authors:** Taylor Sellers, Moleca Ghannam, Kojo Asantey, Jennifer Klei, Elizabeth Olive, Victoria Roach

**Affiliations:** 1 Medical Student, Oakland University William Beaumont School of Medicine; 2 Medical Student, Student, Oakland University William Beaumont School of Medicine; 3 Assistant Professor of Anatomy, Department of Foundational Medical Studies, Oakland University William Beaumont School of Medicine

**Keywords:** Surgery, Surgical Skills, Laparoscopy, Minimally Invasive Surgery, Laparoscopic Skills

## Abstract

**Introduction:**

Despite the increasing prevalence of minimally invasive surgery (MIS), medical students receive little training in MIS techniques like laparoscopy. Cost is perhaps the biggest obstacle, as expensive laparoscopic skill simulators (box trainers) are needed to mimic the laparoscopic environment. Low-cost, homemade box trainers have been built and described in the literature but are generally relegated to self-directed practice for surgical residents. These do-it-yourself (DIY) box trainers are uniquely capable of addressing cost as a major barrier to laparoscopic skills training for medical students but have not previously been used specifically for this purpose.

**Methods:**

Students from the Oakland University William Beaumont School of Medicine (*n* = 17) participated in a laparoscopic skills training course using DIY box trainers. Four basic laparoscopic tasks were taught using live demonstrations followed by directed practice. Learners were instructed to record their first and last attempts in order to enable self-assessment of their progress.

**Results:**

All learners were able to successfully complete each of the four laparoscopic tasks by the end of their respective sessions. Feedback from the learners in the form of a postsession survey indicated that the course provided an enjoyable and high-quality experience.

**Discussion:**

This resource is effective at providing medical students with a low-cost opportunity to experience laparoscopy and develop basic laparoscopic skills.

## Educational Objectives

During this activity, learners will be able to:

1. Familiarize themselves with basic laparoscopic tools, including graspers, scissors, needle drivers, and knot pushers, and how to properly use them.
2. Experience and appreciate the inherent challenges of laparoscopy firsthand.
3. Acquire manual dexterity sufficient to successfully manipulate tools and target structures in a simulated laparoscopic environment.
4. Apply newly acquired knowledge to complete four basic laparoscopic tasks.

## Introduction

Medical students often receive little to no training in laparoscopic skills during their undergraduate medical education. This is in stark contrast to actual surgical practice, which is becoming increasingly reliant on minimally invasive surgical techniques.^1–3^ Ideally, medical schools should be providing students with modern technical skills and experiences, including meaningful exposure to laparoscopic skills, to complement their didactic learning. While laparoscopic skill training for medical students has been studied, it remains rare.^4–6^ This rift between surgical education and surgical practice means students may be graduating from medical school inadequately prepared and poorly informed about not-too-distant career choices.

The cost requirements of implementing a laparoscopic skill training program are not trivial.^7^ Resident-level laparoscopic skill training generally begins with the use of commercially available simulators, commonly referred to as box trainers, that allow residents to practice tasks such as cutting and suturing in a controlled environment without the need for human or animal tissue. Box trainers have been shown to be effective for the development of laparoscopic skills.^8^ However, commercial box trainers cost hundreds or thousands of dollars, rendering them impractical for medical student use. In response, many designs for lower-cost do-it-yourself (DIY) box trainers have surfaced and can be effective for basic skills practice in junior surgical trainees.^9^ These DIY box trainers present a unique solution to the issue of cost and may facilitate more widespread laparoscopic skill training for medical students. However, their de facto purpose has been self-directed practice for residents rather than being considered a premiere tool for undergraduate surgical education.

This resource is aimed at medical school faculty and other program coordinators interested in implementing a low-cost laparoscopic skill program in undergraduate medical education. It consists of plans for building a DIY box trainer including interchangeable modules to facilitate four laparoscopic tasks, videos that demonstrate the tasks for future instructors, and a crash course–style lesson plan to teach the tasks to medical students. The box trainer is a unique design that was deemed capable of facilitating all the planned laparoscopic tasks by general surgeons at William Beaumont Hospital in Royal Oak, Michigan. At a cost of approximately US$50.00, the DIY trainer is an economical alternative to commercial options.^7^

This resource presents a unique opportunity to easily and affordably incorporate a laparoscopic skill curriculum into undergraduate medical education. While DIY box trainers have been built and described previously,^9^ this resource is unique in that it combines detailed instructions for the construction of box trainers with a complementary lesson plan designed for undergraduate medical education that has been successfully tested by medical students.

## Methods

**Figure 1.**
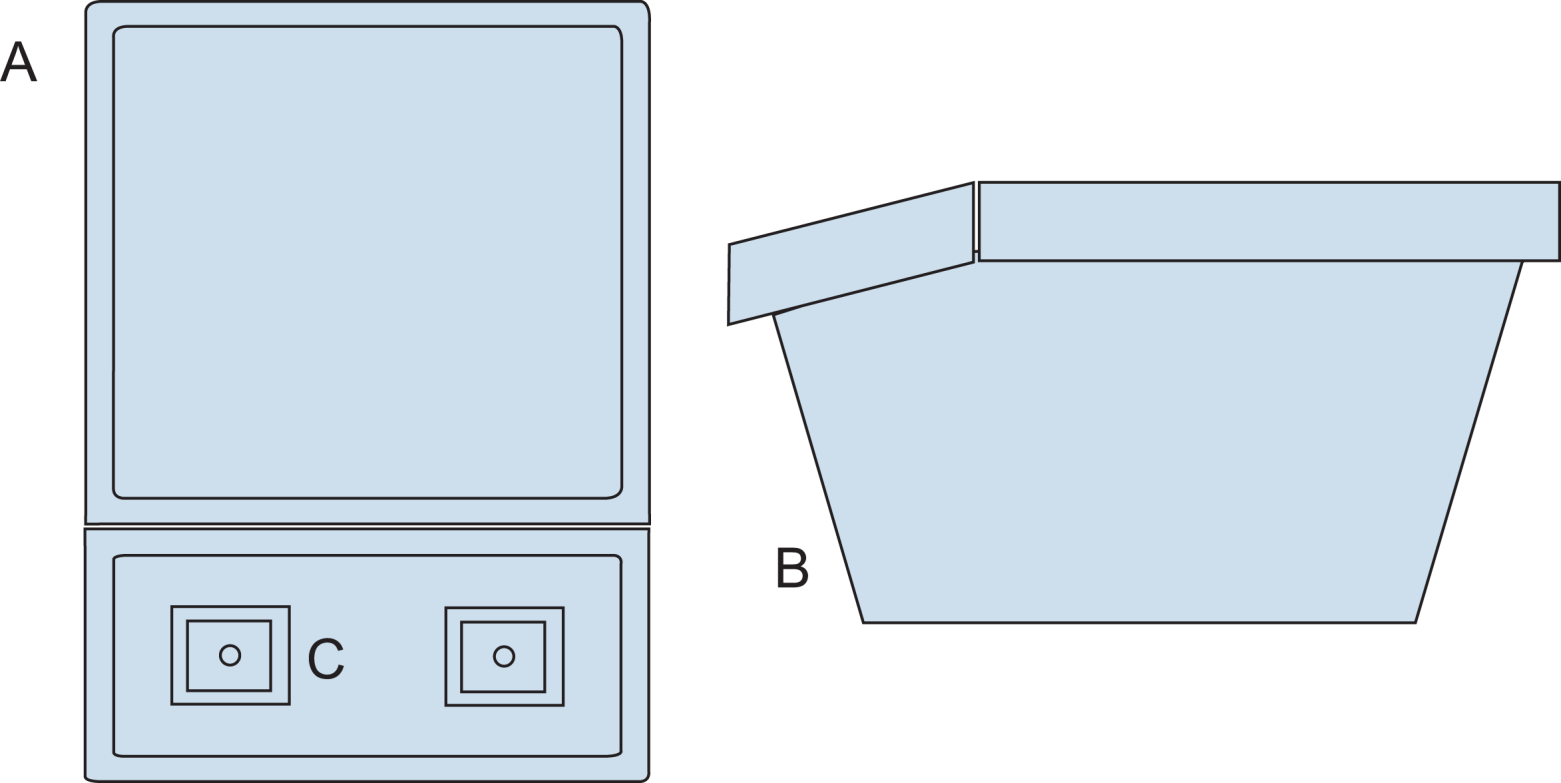
Schematic diagram of the top view (A) and side view (B) of the box trainer described in the resource. The ports for inserting laparoscopic instruments (C) face the learner.

**Figure 2.**
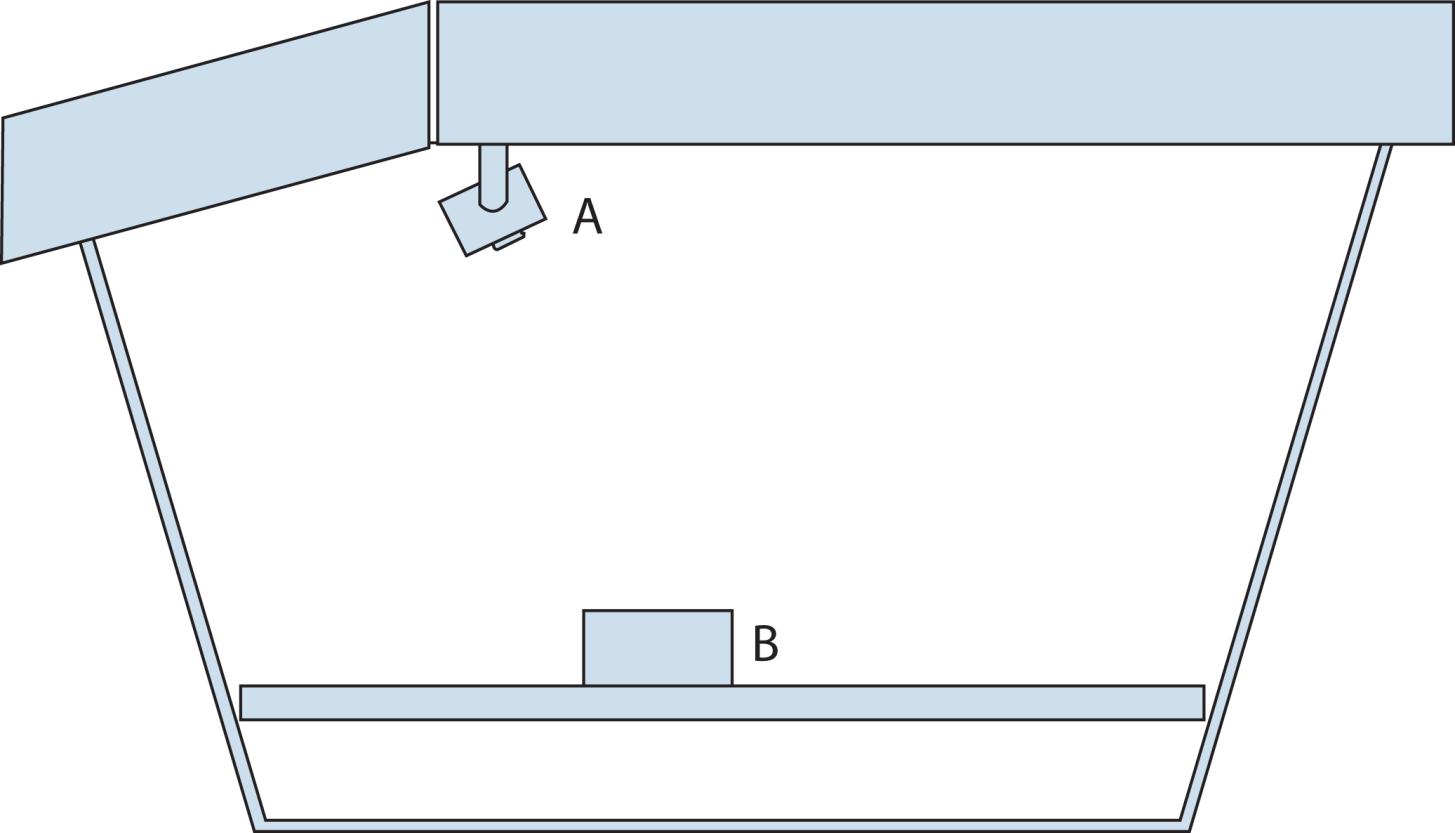
Cutaway schematic diagram showing the interior of the box trainer. The camera module (A) connects to a laptop via USB and displays the box trainer interior. Interchangeable modules (B) allow a variety of tasks to be performed.

Participants were recruited from Oakland University William Beaumont School of Medicine classes of 2021, 2020, and 2019 during their first, second, and third years of medical school, respectively. Prior to the first training session, five box trainers (see Figure 1 & Figure 2) and all associated task sets were constructed and tested using the methods described in Appendices A–D.

The instructor-to-learner ratio for each session was held at 1:5. Learners were not required to have any prerequisite knowledge. The instructor required significant preparation to become proficient enough to perform each task live for the learners with minimal errors. Appendices E–H are instructional videos for each task created after the conclusion of the study to assist future facilitators. Three of the four tasks (precision cutting, intracorporeal suture, and extracorporeal suture) are used as part of the Fundamentals of Laparoscopic Surgery program from the Society of American Gastrointestinal and Endoscopic Surgeons.^10^ The fourth task, thread transfer, is used as part of the SurgTrac training program from eoSurgical.^11^

**Figure 3.**
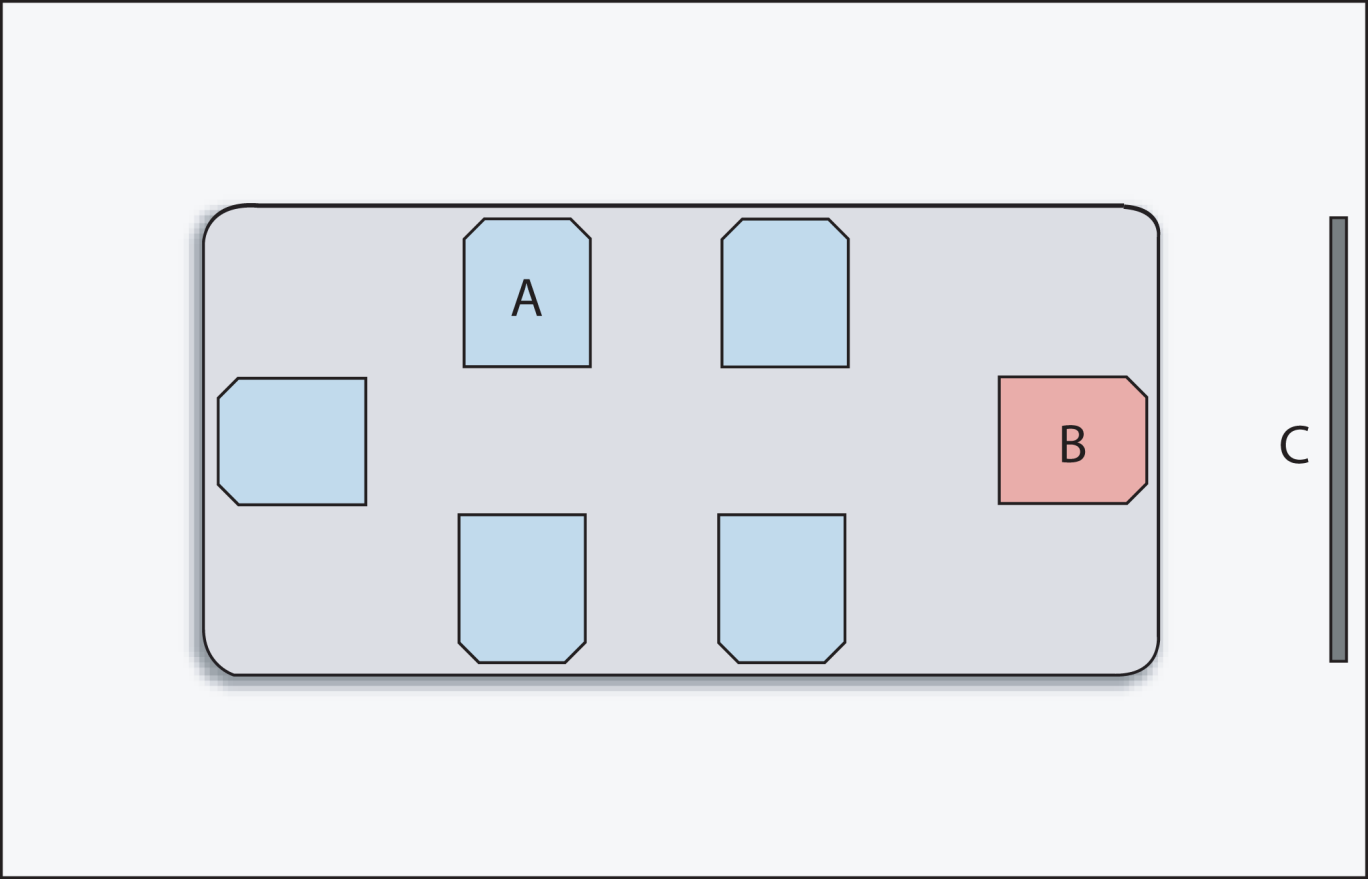
Schematic of the room layout for each learning session. Learner stations (A) and an instructor station (B) were arranged on a conference room table. A television (C) was mounted to the wall and connected to the instructor's box trainer, allowing learners to view live task emonstrations.

On the day of each session, setting up the learner stations required approximately 30 minutes. Five stations were set up on a large conference room table (Figure 3). At each station, a box trainer was placed where the learner would stand. To the left of the box trainer, the swappable task sets were lined up along with gauze for the precision cutting task and sutures for the suturing tasks. To the right of the box trainer, two laparoscopic graspers, one laparoscopic scissors, two laparoscopic needle drivers, and a knot pusher were placed. The session room included a large television, which was used to display the interior of the instructor's box during live demonstrations.

Once the learners arrived, 15 minutes were allocated to explain the format of the session and to ensure that all learners were able to connect the cameras to their laptops. Students were also taught how to record their attempts on their personal laptops. All students at the Oakland University William Beaumont School of Medicine are provided with an Apple MacBook Pro upon matriculation. These were used for the session. QuickTime Player (preinstalled on each MacBook) was used to view the camera feed and record videos. The cameras are compatible with other operating systems, and comparable software can be found on those platforms.

A lesson plan for the sessions can be found in Appendix J. The session was split into four sections, one for each task. Each of the four sections followed a nearly identical format. A live demonstration of the task was performed by the instructor and displayed for the learners on the television screen. During the demonstration, the instructor talked through each step, explained common pitfalls, answered questions, and repeated any portion of the task requested by the learners. After the demonstration, the learners individually completed their first attempt at the task without assistance from the instructor, recording the attempt on their laptops. For the remainder of the time allotted for the section, learners practiced the task with assistance from the instructor as needed. Practice attempts were not recorded. With approximately 5 minutes remaining in the section, learners were instructed to complete and record their final attempt. Participants had the opportunity to view their videos later to reflect on their progress. Each task lasted approximately 50 minutes. The total session time was 4 hours.

The first task was the thread transfer task, which required learners to pass a length of cord (thread) through a series of five rings in a specific sequence (Appendix E). The second task was the precision cutting task, which required learners to cut between two concentric circles drawn on a piece of gauze (Appendix F). The third task was the extracorporeal suturing task, which required learners to place an untrimmed suture through two predrawn dots on either side of a slit cut into a segment of Penrose drain. The suture was then drawn out of the box, and a surgical square knot (three throws) was tied using a knot pusher (Appendix G). The fourth and final task was the intracorporeal suturing task, which required learners to place a trimmed (6-inch) suture through two predrawn dots on either side of a slit cut into a segment of Penrose drain. A surgeon's knot (three throws) was then tied using laparoscopic instruments (Appendix H). Learners were asked to complete a brief Likert scale survey following the session (Appendix K).

## Results

During the training program every participant was able to successfully complete each of the four tasks, confirmed by viewing the recordings of their final attempts. Success was defined as the ability to complete each task from start to finish without assistance from the instructor. Successful completion was predicated on meeting the stated educational objectives.

The Likert scale survey indicated that the experience was enjoyable and successful. In response to the survey, 88% strongly agreed and the remaining 12% somewhat agreed when asked if the DIY box trainer provided a high-quality experience. When students were asked if they enjoyed using the DIY trainer, 94% strongly agreed, and the remaining 6% somewhat agreed. Detailed responses from all the participants are shown in the Table.

**Table.** Results From the Likert Scale Surveys (*N* = 17)

Question	No. (%)				
	Strongly Agree	Somewhat Agree	Neither Agree nor Disagree	Somewhat Disagree	Strongly Disagree
1. The laparoscopic skills trainer was easy to use.	5 (29.41%)	8 (47.06%)	2 (11.76%)	2 (11.76%)	0 (0.00%)
2. The laparoscopic skills trainer was poorly designed.	0 (0.00%)	0 (0.00%)	2 (11.76%)	3 (17.65%)	12 (70.59%)
3. The laparoscopic skills trainer provided a high-quality experience.	15 (88.24%)	2 (11.76%)	0 (0.00%)	0 (0.00%)	0 (0.00%)
4. I enjoyed using the laparoscopic skills trainer.	16 (94.12%)	1 (5.88%)	0 (0.00%)	0 (0.00%)	0 (0.00%)
5. The quality of the laparoscopic skills trainer made the tasks more difficult than they should have been.	0 (0.00%)	3 (17.65%)	1 (5.88%)	6 (35.29%)	7 (41.18%)
6. A higher quality trainer would have provided a better experience.	2 (11.76%)	4 (23.53%)	3 (17.65%)	2 (11.76%)	6 (35.29%)
7. The laparoscopic skills trainer worked as expected.	12 (70.59%)	2 (11.76%)	2 (11.76%)	1 (5.88%)	0 (0.00%)

## Discussion

While the design for our box trainers was more elaborate than many existing ones,^9^ the intention was to create a box trainer that achieved parity with commercial box trainers with respect to functionality and user experience. It was also important to use parts and hardware that were standardized and widely available throughout the United States whenever possible, as the intent was for the box trainers to be easily reproducible. For example, a recycling bin from IKEA was used as a base due to the static nature of its product lines relative to other national retailers, and screws, washers, nuts, and other hardware were standard sizes available at any hardware or home improvement store. The only power tools needed for construction were a drill and a rotary tool, both widely available and inexpensive. The author who designed and built the box trainers had no professional or even hobbyist experience in engineering, construction, or building. It is expected that the box trainers will be reproducible by laypersons.

The demand to participate in this experience was high. Though only a small number of slots were available, recruitment efforts were not capped due to uncertainty about the potential demand. A total of 102 students elected to complete the consent process, and the 20 available slots were filled within 45 minutes of online sign-up opening. Three participants were lost due to no-shows, resulting in 17 total participants. The high demand to participate was particularly significant when considering that the study population consisted of busy medical students. Sessions were taught on Saturday mornings, lasted 4 hours, and offered no incentives to participate beyond the experience itself. These particular medical students were also inundated with requests to participate in studies due to a research requirement for graduation. The popularity of the program is thus very telling and suggests that students are hungry for hands-on experiences in surgery and that those experiences are few and far between.

The biggest limitation of this resource is access to laparoscopic instruments. They are expensive, and at present, no low-cost alternatives exist. The instruments must either be borrowed or donated, and programs may struggle to secure sufficient access. Each learner must also supply his or her own laptop, though laptops are significantly more common in comparison. Finally, access to a projector or television screen is required for live demonstrations. Price fluctuations and bulk pricing effects are also a potential limitation to implementing the resource. In this instance, the final cost of materials for the box trainers was US$48.54 each (before tax). The materials to create a full set of task modules (thread transfer, precision cutting, and suture block) for each trainer were an additional US$10.44 each (before tax). It is important to note that these prices were for materials purchased in quantities sufficient to build five box trainers. Because of bulk pricing, the cost per trainer will decrease or increase if more than five or fewer than five box trainers, respectively, are built. Consumable supplies (sutures, gauze squares, and Penrose drains) were not included in price calculations because they correlate with the number of learners rather than the number of box trainers. Consumable supply needs will therefore vary considerably depending on the needs of the organization implementing the program. A list with hyperlinks to the exact materials we used can be found in Appendix I.

While the resource was well received, there are also limitations in the outcomes assessment. First, the sample size was small. Second, it is likely that the final cohort comprised students who had an interest in minimally invasive surgery due to selection bias. Finally, outcomes were based on the learners' experience using the DIY box trainer, but given that there were no similar programs in the medical school curriculum, learners lacked experience with a commercially available box trainer for comparison.

In addition, a few technical issues were noted while implementing the resource that could have been prevented. We used 75-cm controlled-release sutures for the suturing tasks. During the extracorporeal suturing task, several learners found that one or both ends fell back into the box trainer at some point because of the depth of the box trainer. While a 75-cm length was sufficient to complete the task, this issue can be addressed in the future by using longer sutures. For the intracorporeal suturing task, some learners had their needles separate from the suture during attempts, requiring them to start over. Controlled-release sutures should not make a difference to a practiced learner with proper technique but were not ideal for a novice learner.

Based on the success of this resource, future research questions, including validation of the DIY box trainer design against commercially available box trainers and exploration of student attitudes toward the use of DIY equipment in undergraduate medical education, will be explored. Future directions could include exploring the potential impact of engagement, satisfaction, and skill acquisition if learners participate in the box trainer construction process, whether or not participation in the program impacts specialty choice, and whether or not students who participate in the program perform better on their surgical clerkships. It is our hope that this research will encourage the introduction of surgical skill training in undergraduate medical curricula.

## Appendices

A. Box Trainer Instructions.docx
B. Thread Transfer Instructions.docx
C. Precision Cutting Instructions.docx
D. Suture Block Instructions.docx
E. Thread Transfer Demonstration Video.mp4
F. Precision Cutting Demonstration Video.mp4
G. Extracorporeal Suture Demonstration Video.mp4
H. Intracorporeal Suture Demonstration Video.mp4
I. Supply List.docx
J. Lesson Plan.docx
K. Postsession Survey.docx All appendices are peer reviewed as integral parts of the Original Publication.
