# Supplementary material for: Low-Cost Laparoscopic Skill Training for Medical Students Using Homemade Equipment
Source: MedEdPORTAL. 2019 Feb 27;15:10810. doi: 10.15766/mep_2374-8265.10810 (PMC6415328; doi:10.15766/mep_2374-8265.10810)
Supplement: Supplementary file 1 — A. Box Trainer Instructions.docx B. Thread Transfer Instructions.docx C. Precision Cutting Instructions.docx D. Suture Block Instructions.docx E. Thread Transfer Demonstration Video.mp4 F. Precision Cutting Demonstration Video.mp4 G. Extracorporeal Suture Demonstration Video.mp4 H. Intracorporeal Suture Demonstration Video.mp4 I. Supply List.docx J. Lesson Plan.docx K. Postsession Survey.docx [file mep-15-10810-s001.zip › A. Box Trainer Instructions.docx]

**Box Trainer**

***Materials***

| Quantity | Description | Approximate Cost |
| --- | --- | --- |
| 1 | IKEA Sortera 10 Gallon Bin With Lid | $10.00 for 1 |
| 1 | 1/2" Thick MDF Panel, Cut to 11 1/4" x 13 1/2" | $12.00 for 6* |
| 4 | Metal Corner Brace - 2 1/2" x 2 1/2" | $14.00 for 40 |
| 1 | Thin Mousepad | $5.00 for 1 |
| 2 | Crimp Ferrule - AWG 1 (11mm inner diameter) | $6.00 for 10 |
| 17 | #6-32 1/2" Machine Screws | $5.00 for 100 |
| 17 | #6-32 1/2" Hex Machine Screw Nut | $5.00 for 100 |
| 16 | #6 Flat Washer | $4.00 for 100 |
| 2 | Rubber O Rings – Inside Diameter: 7mm/0.28”; Outside Diameter: 13mm/0.51”; Thickness: 3mm/0.12” | $6.00 for 50 |
| 1 | USB Camera Module | $20.00 for 1 |
| 5 | Zip Ties | $5.00 for 100 |
| 2 | 2"x4" Velcro Strip (Loop and Hook Sides) | $7.00 for 4 |
| 2’ | 1/8" Paracord | $3.00 for 50' |
| 2 | Alligator Clips | $6.00 for 20 |
| 2 | 12” Long Piece of 2x4 | Variable** |
| 1 | Battery Powered Push Light With Self Stick Adhesive | $10.00 for 5 |

* This price is for a single large sheet of MDF that was cut into six panels

** Home improvement stores often have scrap pieces of 2x4 that they will sell at a discount

***Tools & Equipment***

Power Drill with 9/64” and 3/16” Drill Bits

Rotary Tool with Sanding Band

Hobby Knife

Pliers

Ruler

Pencil

Cut a 10” piece of packing tape and place it on a flat surface sticky-side up. Arrange two corner braces in the shape of a rectangle and place them on the edge of the tape. Do the same to the other edge.

It can be difficult to accurately measure length in the recessed portion of the lid because the sides taper slightly inward as they move away from the hinge. The tape from the previous step will ensure that the two rectangles are the appropriate distance apart. Center the tape/corner brace assembly and tape it to the lid approximately 1 1/4" (approximately one ruler width in this case) from the front edge.

Add additional tape to secure the corner braces to the lid. The holes in the corner braces will be used as a drill guide in the next step.

Using a 9/64” drill bit, drill a hole through the lid in each of the 16 holes in the corner braces. Drill a hole in the center of the square using the largest drill bit you have.

Using a rotary tool with a sanding band, enlarge the center hole within each rectangle. Leave a rim of plastic between the hole and the corner braces. This area will be covered, so the center hole does not need to be precisely measured or perfectly shaped.

Once all holes are drilled, remove the tape and the corner braces.

On the other side of the hinge, make a mark 6 1/2" from the recess sidewall (the total length is 13” so the measurement can be made from either side). The mark should be about 1/4" from the hinge-adjacent sidewall.

Drill a hole at the mark using a 9/64” drill bit.

This is the final view with all holes drilled and all tape removed.

A mouse pad will be used to create the “skin” of the trainer. A thin mouse pad with some capacity for stretch is ideal, as is a fabric top which resists tearing.

If the mouse pad has rounded edges, trim them with scissors or a hobby knife so that they are square.

Cut out two rectangles measuring 2” x 2 1/2". They should be the same size as the rectangle formed by two corner braces.

Tape the mouse pad cutouts with the corner braces on top to a piece of scrap wood or MDF. As with the lid, the corner braces will be used as drill guides to create holes in the mouse pad in the correct locations. Note that the corner braces can be placed in multiple configurations, so make sure that they match the configuration used on the lid.

Drill 16 holes through the mouse pad using a 9/64” drill bit. Remove the tape and mouse pad cutouts.

In place of trocars, crimp ferrules will be used. These ferrules are AWG 1 with a diameter of 11mm (measured at the metal portion).

Place the ferrule in the center of mouse pad and cut around it using a hobby knife. Discard the circle cutout.

Assemble the mouse pad, corner brace, and ferrule apparatus on the lid. Place 6/32” x 1/2" machine screws through each of the holes.

On the backside, secure the machine screws using #6 flat washers followed by 6/32” hex machine screw nuts.

Place a rubber O ring around the ferrule and slide it up to the mouse pad. This will hold the ferrule in place.

Attach the camera module to the underside of the lid using the same size machine screw, washer, and nut as the corner braces.

Unlike the corner braces, the nut and washer should be on the outside of the lid. If the nut and washer are on the underside, the shaft of the machine screw will prevent the proper placement of the camera.

Loosen the screws on the side of the camera mounting bracket and slide the camera module closer to the lid. Tighten the bracket screws.

Position the cord of the camera so that it runs toward the back of the lid. Tape it in position with packing tape.

Drill four sets of holes on either side of the cord using a 9/64” drill bit. Remove the tape.

Use zip ties to tie down the cable. Trim the excess.

Using a rotary tool, make a notch in the back of the lid so the cord can exit.

Drill two holes on either side of the notch above the level of the cord using a 9/64” drill bit. Place a zip tie through the holes to close off the notch. Trim the excess.

Place a push light on the underside of the lid a few inches from the camera. The lid is now complete. Set it aside.

Place two Velcro strips (hook side) on the MDF panel forming a large square. They should be placed 1 1/4" from the front edge (the width of the ruler in this case). There should be 3 1/2" of space on either side of the Velcro square.

Place two pieces of 2x4 in the base of the bin.

Place the MDF panel in the bin on top of the 2x4 pieces.

Measuring from the front edge of the MDF panel, use a pencil to make two marks on the wall of the bin at 1” and 2”. The pencil should be laying flat against the MDF.

Do the same to the other side.

Remove the MDF panel and 2x4 pieces and drill holes at each of the four marks using a 3/16” drill bit.

Cut two pieces of paracord approximately 1’ long. They will be trimmed to the correct length once put in place. Two alligator clips will also be needed.

Feed one end of the paracord into the alligator clip and crimp it in place using pliers. Do this for both pieces of paracord.

Place the 2x4 pieces and MDF panel back in the box. Starting from the inside of the box, feed the free end of the paracord through the hole closest to the front of the box. Feed the end back into the box using the second hole. Repeat this process on the other side.

Attach a clip and gauze assembly (see Precision Cutting instructions) to the Velcro square. Attach both alligator clips to the gauze. Use this setup to pull both paracords taut, ensuring that they are approximately equal in length and the gauze does not pull unevenly to one side.

Tie knots in the back portions of the paracord as close to the wall of the box as possible.

Trim the paracord close to the knot and burn the end to prevent fraying. Repeat this process on the other side.

Place the lid on top of the box. The cord for the camera will prevent it from closing completely. Using a pencil, make a mark on the box in the shape of the notch in the lid.

Using a rotary tool with a sanding band, make a notch for the cord in the rim of the box using the mark made in the previous step.

The lid should now fit flush to the box. The box trainer is now complete.
