# Supplementary material for: Low-Cost Laparoscopic Skill Training for Medical Students Using Homemade Equipment
Source: MedEdPORTAL. 2019 Feb 27;15:10810. doi: 10.15766/mep_2374-8265.10810 (PMC6415328; doi:10.15766/mep_2374-8265.10810)
Supplement: Supplementary file 1 — A. Box Trainer Instructions.docx B. Thread Transfer Instructions.docx C. Precision Cutting Instructions.docx D. Suture Block Instructions.docx E. Thread Transfer Demonstration Video.mp4 F. Precision Cutting Demonstration Video.mp4 G. Extracorporeal Suture Demonstration Video.mp4 H. Intracorporeal Suture Demonstration Video.mp4 I. Supply List.docx J. Lesson Plan.docx K. Postsession Survey.docx [file mep-15-10810-s001.zip › B. Thread Transfer Instructions.docx]

**Thread Transfer**

***Materials***

| Quantity | Description | Approximate Cost |
| --- | --- | --- |
| 5 | #10 Screw Eyes | $5.00 for 50 |
| 11" | 1/8" Paracord | $3.00 for 50' |
| 1 | 2"x4" Velcro Strip (Loop Side) | $7.00 for 4 |
| 1 | 1x3 Board Cut to 6" Length | $10.00 for 8' |

***Tools & Equipment***

Drill Press or Hand Drill with Drill Guide – 1/8” Drill Bit

Ruler

Pencil

Measure 1” in from either side of the board and make a vertical line with a pencil.

Within the space formed by the two vertical lines, draw two diagonal lines to form an “X”.

Draw two additional vertical lines 1/4" inward from the original vertical lines.

Drill five holes where the previously drawn lines cross using a 1/8” drill bit.

Screw the screw eyes into the holes. The height of the screw eyes should gradually decrease from the back to the front.

Place a Velcro strip (loop side) on the backside of the block.

Cut an 11” segment of paracord. Tape and/or burn the edges to prevent fraying.

The thread transfer set is complete.
