# Supplementary material for: Low-Cost Laparoscopic Skill Training for Medical Students Using Homemade Equipment
Source: MedEdPORTAL. 2019 Feb 27;15:10810. doi: 10.15766/mep_2374-8265.10810 (PMC6415328; doi:10.15766/mep_2374-8265.10810)
Supplement: Supplementary file 1 — A. Box Trainer Instructions.docx B. Thread Transfer Instructions.docx C. Precision Cutting Instructions.docx D. Suture Block Instructions.docx E. Thread Transfer Demonstration Video.mp4 F. Precision Cutting Demonstration Video.mp4 G. Extracorporeal Suture Demonstration Video.mp4 H. Intracorporeal Suture Demonstration Video.mp4 I. Supply List.docx J. Lesson Plan.docx K. Postsession Survey.docx [file mep-15-10810-s001.zip › C. Precision Cutting Instructions.docx]

**Precision Cutting**

***Materials***

| Quantity | Description | Approximate Cost |
| --- | --- | --- |
| 1 | 6" Chip Bag Clip | $5.00 for 4 |
| 2" | Velcro Tape (Loop Side) | $3.00 for 3' |
| 1 | 4"x4" Gauze Squares | $5.00 for 200 |

***Tools & Equipment***

Circle Stencil with 2 1/4" Diameter and 2 1/2" Diameter Cutouts

Scissors

Felt Tip Marker

Start with one gauze square.

If the gauze is 4-ply, unfold it and cut it in half to create two 2-ply squares.

Using the 2 1/4" diameter circle stencil, draw a circle in the center of the gauze.

Use a light touch to avoid dragging the gauze along with the pen. Touch up any gaps in the circle with the marker.

Using the 2 1/4" diameter stencil, draw another circle around the first.

This process will need to be completed multiple times to allow repeat attempts at the task.

Place a strip of Velcro (loop side) on the back of a chip bag clip.

The clip and gauze assembly is complete.
