# Supplementary material for: Low-Cost Laparoscopic Skill Training for Medical Students Using Homemade Equipment
Source: MedEdPORTAL. 2019 Feb 27;15:10810. doi: 10.15766/mep_2374-8265.10810 (PMC6415328; doi:10.15766/mep_2374-8265.10810)
Supplement: Supplementary file 1 — A. Box Trainer Instructions.docx B. Thread Transfer Instructions.docx C. Precision Cutting Instructions.docx D. Suture Block Instructions.docx E. Thread Transfer Demonstration Video.mp4 F. Precision Cutting Demonstration Video.mp4 G. Extracorporeal Suture Demonstration Video.mp4 H. Intracorporeal Suture Demonstration Video.mp4 I. Supply List.docx J. Lesson Plan.docx K. Postsession Survey.docx [file mep-15-10810-s001.zip › D. Suture Block Instructions.docx]

**Suture Block**

***Materials***

| Quantity | Description | Approximate Cost |
| --- | --- | --- |
| 1 | 1x3 Board Cut to 6" Length | $10.00 for 8' |
| 2 | 3"x3"x1/4" Foam Rubber Padding | $10.00 for 16 |
| 1 | Craft Foam Sheet | $1.00 for 1 |
| 1 | 2"x4" Velcro Strip (Loop Side) | $7.00 for 4 |
| 6" | White Velcro Tape (Loop Side) | $3.00 for 3' |
| 1 | 1/2" Diameter Penrose Drain | $14.00 for 25 |
| 1" | Velcro Tape (Hook Side) | $3.00 for 3' |

***Tools & Equipment***

Hobby Knife

Ruler

Scissors

Felt Tip Marker

Attach foam rubber padding to the wooden block using the self-adhesive backing or contact cement.

Trim the excess foam rubber from the block using a hobby knife.

Attach a layer of craft foam using self-adhesive backing or contact cement. Use a color that will contrast well with the needle and suture material, such as red.

Attach a strip of Velcro (loop side) to the craft foam. It is important that the Velcro is white rather than black, as black suture material will not appear clearly on camera against a black background.

Attach a 2”x4” Velcro strip (loop side) to the back of the block.

Pass a Penrose drain under water to remove any powder from the exterior and dry it with a towel.

Cut a 1” segment from the Penrose drain.

Using scissors, cut a small curved piece from the crease in the Penrose drain.

Flatten the Penrose drain segment so that the cutout is in the center. Draw two dots on either side of the cutout with a felt tip marker. Dots should be approximately 2mm from the opening and approximately 2mm in diameter.

Cut a 1” segment from a strip of Velcro tape (hook side). Then cut that segment in half lengthwise.

Remove the plastic backing from one of the Velcro pieces. Place the crease of the Penrose drain in the center.

Flatten the Penrose drain against the Velcro so that the entire adhesive surface makes contact.

The Velcro holds the Penrose drain open. Prepare more Penrose drain segments as needed.

The suture block assembly is complete.
