# Supplementary material for: Low-Cost Laparoscopic Skill Training for Medical Students Using Homemade Equipment
Source: MedEdPORTAL. 2019 Feb 27;15:10810. doi: 10.15766/mep_2374-8265.10810 (PMC6415328; doi:10.15766/mep_2374-8265.10810)
Supplement: Supplementary file 1 — A. Box Trainer Instructions.docx B. Thread Transfer Instructions.docx C. Precision Cutting Instructions.docx D. Suture Block Instructions.docx E. Thread Transfer Demonstration Video.mp4 F. Precision Cutting Demonstration Video.mp4 G. Extracorporeal Suture Demonstration Video.mp4 H. Intracorporeal Suture Demonstration Video.mp4 I. Supply List.docx J. Lesson Plan.docx K. Postsession Survey.docx [file mep-15-10810-s001.zip › I. Supply List.docx]

| Recycling Bin - <http://www.ikea.com/us/en/catalog/products/10255897/> | |
| --- | --- |
|  | *IKEA Sortera 10 Gallon Bin With Lid* |
| Camera Module - <http://a.co/87BHQe3> | |
|  | *ELP Mini USB Camera Module* |
| Machine Screws - <http://a.co/6IqUYxJ> | |
|  | *#6-32 1/2" Machine Screws* |
| Machine Nuts - <http://a.co/hJrYsUb> | |
|  | *#6-32 1/2" Hex Machine Screw Nut* |
| Washers - <http://a.co/8NinDjO> | |
|  | *#6 Flat Washer* |
| Corner Braces - <http://a.co/8zQuaMr> | |
|  | *2.5"x 2.5" Metal Corner Braces* |
| Crimp Ferrules - <http://a.co/eNvCNdE> | |
|  | *AWG 1 (11mm Inner Diameter)* |
| Velcro Strips - <http://a.co/j7MJIjF> | |
|  | *2"x4" Strips* |
| Alligator Clips - <http://a.co/65kTOSA> | |
|  | *2" x 0.4" x 0.2" Clips* |
| Penrose Drains - <http://a.co/hkkAsCh> | |
|  | *0.5" Diameter* |
| Gauze Squares - <http://a.co/fXL0lXR> | |
|  | *4"x4" 2-ply, nonwoven* |
| Chip Clips - <http://a.co/boGxvAl> | |
|  | *6" Wide* |
| Velcro Tape - <http://a.co/f68OxUS> | |
|  | *3/4" Wide* |
| Foam Padding - <http://a.co/aOtUPBa> | |
|  | *Acoustic Damper Anti-Vibration Closed-Cell Pads w/Adhesive, 3"x3"x1/4"* |
| Mouse Pad - <http://a.co/aNiF9aT> | |
|  | *7.5"x 8"x0.12"* |
| O Rings - <http://a.co/3BemURJ> | |
|  | *ID: 7mm/0.28'', OD: 13mm/0.51'', Thickness: 3mm/0.12''* |
| Adhesive Foam Sheets - <http://www.michaels.com/creatology-value-pack-adhesive-foam-sheets/10104248.html> | |
|  | *4" x 6" Self Adhesive Foam Sheets* |
| Screw Eyes - <https://www.homedepot.com/p/Everbilt-10-Zinc-Plated-Steel-Screw-Eye-50-Pack-803212/204273861> | |
|  | *#10 Zinc Plated Screw Eyes* |
| Paracord - <https://www.homedepot.com/p/Everbilt-Assorted-Colors-1-8-in-x-50-ft-Para-cord-12715/203602865> | |
|  | *1/8 in. x 50 ft.* |

The research team does not endorse any specific manufacturers, suppliers, or retailers. All links were confirmed active as of January 2018. Prices are subject to fluctuation.
