# Supplementary material for: Low-Cost Laparoscopic Skill Training for Medical Students Using Homemade Equipment
Source: MedEdPORTAL. 2019 Feb 27;15:10810. doi: 10.15766/mep_2374-8265.10810 (PMC6415328; doi:10.15766/mep_2374-8265.10810)
Supplement: Supplementary file 1 — A. Box Trainer Instructions.docx B. Thread Transfer Instructions.docx C. Precision Cutting Instructions.docx D. Suture Block Instructions.docx E. Thread Transfer Demonstration Video.mp4 F. Precision Cutting Demonstration Video.mp4 G. Extracorporeal Suture Demonstration Video.mp4 H. Intracorporeal Suture Demonstration Video.mp4 I. Supply List.docx J. Lesson Plan.docx K. Postsession Survey.docx [file mep-15-10810-s001.zip › J. Lesson Plan.docx]

**Preparation:**

- Familiarize yourself with this lesson plan.
- Practice each of the four tasks until you are comfortable performing each of them live for learners. Instructional videos for each task can be found in Appendix E through Appendix H.
- Pre-record a successful attempt at each task to be played on a loop while the learners practice.
- Supplies (per learner):
  - (1) Laptop computer (provided by student)
  - (1) Training box (see Appendix A)
  - (1) Thread transfer set (see Appendix B)
  - (1) Precision cutting set (see Appendix C)
  - (1) Suture block set (see Appendix D)
  - (2) Laparoscopic graspers
  - (1) Laparoscopic scissors
  - (2) Laparoscopic needle drivers
  - (1) Knot pusher
  - (Variable) Gauze squares for precision cutting (see Appendix C)
  - (Variable) Penrose drains for suturing (see Appendix D)
  - (Variable) Sutures (26mm ½ circle, at least 75mm in length)
  - Sharps disposal
  - Projector or monitor for live demonstrations
  - Total time: 4 hours

**Goals:**

During this activity, learners will be able to:

1. Experience the inherent challenges of laparoscopy first hand.
2. Acquire manual dexterity sufficient to successfully manipulate objects in a simulated laparoscopic environment.
3. Apply laparoscopic skills to complete four basic laparoscopic tasks.

**Learning Objectives:**

The learner will:

1) Demonstrate acquired laparoscopic skills by successfully completing four laparoscopic tasks.
   a. Thread Transfer (50 minutes)
      i. Supplies (per learner):
         1. Training box, 2x laparoscopic graspers, thread transfer set
      ii. Teaching flow:
          1. Ensure that all learners are able to successfully able to connect the camera module to their laptops and view the inside of the box.
          2. Perform a live demonstration of the thread transfer task using a projector or monitor to display the contents of one of the training boxes.
          3. Allow the learners to perform their first attempt at the task without assistance while recording the attempt on their own laptops.
          4. Allow the learners to practice the task while a pre-recorded video of the task plays on the projector or monitor until five minutes remain in the allotted time. Provide assistance and additional demonstrations as needed.
          5. Instruct the learners to perform and record their final attempt using the final five minutes.
   b. Precision Cutting (50 minutes)
      i. Supplies (per learner):
         1. Training box, laparoscopic grasper, laparoscopic scissors, precision cutting set, gauze squares with prepared circles (as many as the learner needs)
      ii. Teaching flow:
          1. Perform a live demonstration of the precision cutting task using a projector or monitor to display the contents of one of the training boxes.
          2. Allow the learners to perform their first attempt at the task without assistance while recording the attempt on their own laptops. Instruct learners to keep their first gauze cutout to compare to their final cutout.
          3. Allow the learners to practice the task while a pre-recorded video of the task plays on the projector or monitor until five minutes remain in the allotted time. Provide assistance and additional demonstrations as needed.
          4. Instruct the learners to perform and record their final attempt using the final five minutes. Instruct learners to keep their final gauze cutout to compare to their first cutout.
   c. Extracorporeal Knot (75 minutes)
      i. Supplies (per learner):
         1. Training box, 2x laparoscopic needle drivers, suture block set, Penrose drains (as many as the learner needs), sutures (as many as the learner needs)
      ii. Teaching flow:
          1. Perform a live demonstration of the extracorporeal suture task using a projector or monitor to display the contents of one of the training boxes.
          2. Allow the learners to perform their first attempt at the task without assistance while recording the attempt on their own laptops. Instruct learners to keep their first Penrose drain to compare to their final Penrose drain.
          3. Allow the learners to practice the task without recording while a pre-recorded video of the task plays on the projector or monitor until five minutes remain in the allotted time. Provide assistance and additional demonstrations as needed.
          4. Instruct the learners to perform and record their final attempt using the final five minutes. Instruct learners to keep their final Penrose drain to compare to their first Penrose drain.
   d. Intracorporeal Knot (50 minutes)
      i. Supplies (per learner):
         1. Training box, 2x laparoscopic needle drivers, suture block set, Penrose drains (as many as the learner needs), sutures (as many as the learner needs)
      ii. Teaching flow:
          1. Perform a live demonstration of the extracorporeal suture task using a projector or monitor to display the contents of one of the training boxes.
          2. Allow the learners to perform their first attempt at the task without assistance while recording the attempt on their own laptops. Instruct learners to keep their first Penrose drain to compare to their final Penrose drain.
          3. Allow the learners to practice the task without recording while a pre-recorded video of the task plays on the projector or monitor until five minutes remain in the allotted time. Provide assistance and additional demonstrations as needed.
          4. Instruct the learners to perform and record their final attempt using the final five minutes. Instruct learners to keep their final Penrose drain to compare to their first Penrose drain.
2) Perform self-assessment and reflection
   a. Instruct learners to review the videos they recorded of first and last attempts for each task, as well as their gauze cutouts and Penrose drain.
   b. Encourage them to make note of the reduction in time needed to complete each task, the fluidity and economy of their movements, the reduction in the number of deviations outside the lines in the precision cutting task, and the integrity of their knots.
