# Supplementary material for: Low-Cost Laparoscopic Skill Training for Medical Students Using Homemade Equipment
Source: MedEdPORTAL. 2019 Feb 27;15:10810. doi: 10.15766/mep_2374-8265.10810 (PMC6415328; doi:10.15766/mep_2374-8265.10810)
Supplement: Supplementary file 1 — A. Box Trainer Instructions.docx B. Thread Transfer Instructions.docx C. Precision Cutting Instructions.docx D. Suture Block Instructions.docx E. Thread Transfer Demonstration Video.mp4 F. Precision Cutting Demonstration Video.mp4 G. Extracorporeal Suture Demonstration Video.mp4 H. Intracorporeal Suture Demonstration Video.mp4 I. Supply List.docx J. Lesson Plan.docx K. Postsession Survey.docx [file mep-15-10810-s001.zip › K. Postsession Survey.docx]

For each statement, choose the option that best characterizes your experience with the laparoscopic skills box trainer.

|  | Strongly Agree | Agree | Undecided | Disagree | Strongly Disagree |
| --- | --- | --- | --- | --- | --- |
| The laparoscopic skills trainer was easy to use. | 1 | 2 | 3 | 4 | 5 |
| The laparoscopic skills trainer was poorly designed and/or made. | 1 | 2 | 3 | 4 | 5 |
| The laparoscopic skills trainer provided a high quality experience. | 1 | 2 | 3 | 4 | 5 |
| I enjoyed using the laparoscopic skills trainer. | 1 | 2 | 3 | 4 | 5 |
| The quality of the laparoscopic skills trainer made the tasks more difficult than they should have been. | 1 | 2 | 3 | 4 | 5 |
| A higher quality trainer would have provided a better experience. | 1 | 2 | 3 | 4 | 5 |
| The laparoscopic skills trainer worked as expected. | 1 | 2 | 3 | 4 | 5 |
